# Pain hypervigilant and dissociative profiles of suicide risk among emerging adults

**DOI:** 10.1192/j.eurpsy.2026.10216

**Published:** 2026-07-31

**Authors:** F. Bianco, D. L. Romano, C. Gramaglia, P. Zeppegno, F. Madeddu, R. Calati

**Affiliations:** 1 Department of Psychology, University of Milano-Bicocca, Milan; 2 Department of Translational Medicine, University of Eastern Piedmont; 3 Psychiatry Unit, Maggiore della Carità University Hospital, Novara, Italy; 4 Nîmes University Hospital, Nîmes, France

## Abstract

**Introduction:**

Ideation-to-action theories posit that increased pain tolerance contributes to suicide capability. In a previous theoretical contribution, we revisited this hypothesis in light of evidence on interoceptive functioning, particularly dissociation-induced hypoalgesia and inflammation-induced hypersensitivity. We also hypothesized the existence of two subtypes of individuals at suicide risk: a dissociative vs. a pain hypersensitive, characterized by opposite profiles of interoceptive functioning and potentially different trajectories toward suicide attempt.

**Objectives:**

The aim of the present study was to identify profiles of interoceptive functioning, pain sensitivity and dissociative detachment, and to examine their associations with suicide-related phenomena, namely suicidal ideation (SI), suicidal planning (SP), non-suicidal self-injury (NSSI) and suicide attempt (SA).

**Methods:**

A sample of university students (N=1,420) aged 18-29 completed the Dissociative Experiences Scale (DES-II), a Visual Analogue Scale of Physical Pain (PPVAS), the Pain Vigilance and Awareness Questionnaire (PVAQ), the Multidimensional Assessment of Interoceptive Awareness (MAIA-2) and an ad hoc questionnaire on suicide-related phenomena. A Latent Profile Analysis (LPA) was conducted on MAIA-not-distracting, MAIA-not-worrying, VAS physical pain, PVAQ-awareness, PVAQ-vigilance and DES-depersonalization/derealization. Clustering solutions were compared by fit indices, and profiles were entered into logistical regressions predicting suicide-related phenomena.

**Results:**

The LPA revealed four profiles: 1) *‘Healthy’* (54% of total): good interoceptive functioning, low physical pain, low pain awareness/vigilance and low dissociative detachment; 2) *‘Hypervigilant’* (27%): tendency to worry about pain, high physical pain and pain awareness/vigilance, but low dissociation; 3) *‘Dissociative-hypervigilant’* (10%): tendency to worry about pain, high physical pain and pain awareness/vigilance, and high dissociation; 4) *‘Dissociative-hypovigilant’* (9%): tendency to distract from pain, high physical pain, but low pain awareness/vigilance and high dissociation (Image 1). Compared with profile 1, profiles 3 and 4 were associated with increased odds of current SI (OR=3.4; 5.2), history of SP (OR=2.2; 1.9), of NSSI (OR=3.2; 3.7) and of SA (OR=3.2; 5.4) (Image 2).

**Image 1:**
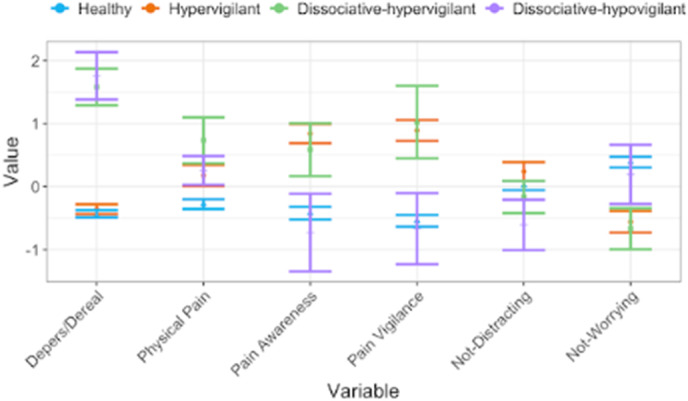
Image 1: Long description.

**Image 2:**
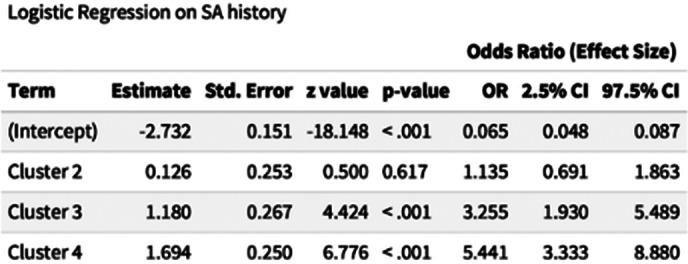
Image 2: Long description.

**Conclusions:**

Our findings highlight that dissociation plays a pivotal role in suicide risk but can manifest along different interoceptive trajectories. Highly dissociative individuals reported above-average pain levels, yet dissociation was linked either to hypervigilance (increased awareness and worry about pain) or to hypovigilance (reduced awareness and tendency to distract from pain). Both dissociative pathways were associated with heightened risk of suicide-related phenomena, though their clinical profiles and risk trajectories appear distinct.

**Disclosure of Interest:**

None Declared

## Long descriptions

### Image 1: Long description

A dot plot with error bars displays standardized values on the y-axis ranging from -1 to 2, against six variables on the x-axis. A legend at the top identifies four groups: Healthy (blue), Hypervigilant (orange), Dissociative-hypervigilant (green), and Dissociative-hypovigilant (purple).

Data trends from left to right:

* Depers/Dereal: Dissociative-hypervigilant and Dissociative-hypovigilant groups show high positive values near 2, while Healthy and Hypervigilant groups are near -0.5.

* Physical Pain: Dissociative-hypervigilant is highest (near 0.8), followed by Dissociative-hypovigilant and Hypervigilant (near 0.4), with Healthy lowest (near -0.2).

* Pain Awareness: Hypervigilant and Dissociative-hypervigilant are highest (0.6 to 0.8), while Dissociative-hypovigilant is lowest (near -0.8).

* Pain Vigilance: Hypervigilant and Dissociative-hypervigilant are highest (0.8 to 1.0), while Dissociative-hypovigilant is lowest (near -0.6).

* Not-Distracting: Hypervigilant is highest (near 0.3), while Dissociative-hypovigilant is lowest (near -0.4).

* Not-Worrying: Healthy is highest (near 0.4), while Dissociative-hypervigilant is lowest (near -0.6).

Navigate back to Image 1:.

### Image 2: Long description

The table is titled Logistic Regression on S A history. It contains eight columns: Term, Estimate, Std. Error, z value, p-value, O R, 2.5% C I, and 97.5% C I. The last three columns are grouped under the header Odds Ratio (Effect Size).

* Row 1: Term is (Intercept). Estimate is -2.732. Std. Error is 0.151. z value is -18.148. p-value is less than .001. O R is 0.065. 2.5% C I is 0.048. 97.5% C I is 0.087.

* Row 2: Term is Cluster 2. Estimate is 0.126. Std. Error is 0.253. z value is 0.500. p-value is 0.617. O R is 1.135. 2.5% C I is 0.691. 97.5% C I is 1.863.

* Row 3: Term is Cluster 3. Estimate is 1.180. Std. Error is 0.267. z value is 4.424. p-value is less than .001. O R is 3.255. 2.5% C I is 1.930. 97.5% C I is 5.489.

* Row 4: Term is Cluster 4. Estimate is 1.694. Std. Error is 0.250. z value is 6.776. p-value is less than .001. O R is 5.441. 2.5% C I is 3.333. 97.5% C I is 8.880.

Navigate back to Image 2:.

